# Development of a cell-based assay to identify hepatitis B virus entry inhibitors targeting the sodium taurocholate cotransporting polypeptide

**DOI:** 10.18632/oncotarget.25348

**Published:** 2018-05-04

**Authors:** Kei Miyakawa, Satoko Matsunaga, Yutaro Yamaoka, Mina Dairaku, Kento Fukano, Hirokazu Kimura, Tomoyuki Chimuro, Hironori Nishitsuji, Koichi Watashi, Kunitada Shimotohno, Takaji Wakita, Akihide Ryo

**Affiliations:** ^1^ Department of Microbiology, Yokohama City University School of Medicine, Kanagawa 236-0004, Japan; ^2^ Isehara Research Laboratory, Technology and Development Division, Kanto Chemical Co., Inc., Kanagawa 259-1146, Japan; ^3^ Department of Virology II, National Institute of Infectious Diseases, Tokyo 162-8640, Japan; ^4^ School of Medical Technology, Faculty of Health Sciences, Gunma Paz University, Gunma 370-0006, Japan; ^5^ Research Center for Hepatitis and Immunology, National Center for Global Health and Medicine, Chiba 272-8516, Japan

**Keywords:** HBV-permissive cell, anti-NTCP monoclonal antibody, glabridin, innate immune signaling, ISG

## Abstract

Sodium taurocholate cotransporting polypeptide (NTCP) is a major entry receptor of hepatitis B virus (HBV) and one of the most attractive targets for anti-HBV drugs. We developed a cell-mediated drug screening method to monitor NTCP expression on the cell surface by generating a HepG2 cell line with tetracycline-inducible expression of NTCP and a monoclonal antibody that specifically detects cell-surface NTCP. Using this system, we screened a small molecule library for compounds that protected against HBV infection by targeting NTCP. We found that glabridin, a licorice-derived isoflavane, could suppress viral infection by inducing caveolar endocytosis of cell-surface NTCP with an IC_50_ of ~40 μM. We also found that glabridin could attenuate the inhibitory effect of taurocholate on type I interferon signaling by depleting the level of cell-surface NTCP. These results demonstrate that our screening system could be a powerful tool for discovering drugs targeting HBV entry.

## INTRODUCTION

Hepatitis B virus (HBV) is the causative agent of chronic hepatitis B (CHB), which can lead to liver cirrhosis and hepatocellular carcinoma. The World Health Organization reported that over 240 million people worldwide have chronic HBV [1]. Despite the effectiveness of the HBV vaccine, worldwide prevalence of the disease remains high, and CHB is a major global health problem. Current therapeutic regimens for CHB include pegylated interferon (IFN) and nucleoside/nucleotide analogues. Both treatments aim to prevent progression of the disease to liver failure, cirrhosis, and hepatocellular carcinoma. However, these treatments have limited effectiveness for HBV clearance [2]. In fact, pegylated IFN maintains viral suppression only in approximately 25% of patients [3]. Nucleoside and nucleotide analogues inhibit HBV replication by targeting viral DNA polymerase, but long-term treatment is required to achieve clinical benefits. For example, a 12-month course of lamivudine achieves clearance of hepatitis B e antigen (HBeAg) in approximately 30% of patients with CHB [4]. Moreover, long-term treatment can be associated with a higher risk of side effects and emergence of drug resistant viruses, resulting in treatment failure and disease progression. Therefore, it is vital to develop new types of antiviral drugs for hepatitis B treatment.

In the HBV life cycle, the hepatitis B surface antigen (HBsAg) initially attaches to heparan sulfate proteoglycans on the host cell surface [5]. This attachment seems to be relatively low affinity and reversible, but is essential for the subsequent more specific interaction between HBs and the hepatocyte-specific bile acid transporter sodium taurocholate cotransporting polypeptide (NTCP) [6]. Although various membrane proteins have been reported to be HBV entry receptors, accumulating evidence now suggests that NTCP is an essential receptor for HBV infection [7]. Discovery of this receptor has dramatically increased our understanding of the molecular basis of HBV entry. Viral entry is currently one of the most important targets in the search for new drugs to treat viral infections and identification of NTCP has kindled interest in exploring compounds that inhibit HBV entry.

NTCP is exclusively expressed at the basolateral membrane of hepatocytes [8–10], and is involved in reuptake of conjugated bile acids from the bloodstream into hepatocytes [11]. NTCP is one of the factors that highly restrict host tropism of HBV to hepatocytes. The specific interaction of the PreS1 domain of HBsAg with NTCP triggers HBV attachment and initiates entry into hepatocytes. The synthetic peptide drug Myrcludex B exhibits significant anti-HBV activity by competitively inhibiting this interaction, both *in vitro* and *in vivo*, and is currently undergoing a phase II clinical trial for chronically HBV-infected patients [12, 13]. Physiological substrates of NTCP such as taurocholic and glycocholic acids can also inhibit HBV infection, suggesting the competitive binding of bile acids and PreS1 to NTCP [14]. Interestingly, PreS1-NTCP binding could trigger type I IFN signaling, whereas bile acid treatment does not [15]. Bile acid intake via NTCP was found to inhibit the IFN pathway in the physiologically relevant range of concentrations [16]. These observations support the possibility that NTCP also takes part in the IFN-mediated innate antiviral response.

Because high expression often causes cell cycle inhibition, there are very few conventional hepatocellular carcinoma cell lines with ectopic expression of high levels of NTCP. Moreover, there are no commercially available monoclonal antibodies (mAbs) specifically recognizing cell-surface NTCP due to the difficulty of producing full-length NTCP protein with native structure. In the current study, we generated HBV-susceptible HepG2 cells with a tetracycline-inducible NTCP gene (iNTCP cells) without affecting cell growth. We also generated a high quality mAb (clone 9A8) to efficiently detect cell-surface NTCP. Using these tools, we identified the compound glabridin, which significantly inhibits HBV infection through the downregulation of NTCP from the cell surface.

## RESULTS

### Preparation of HBV-permissive HepG2 cells with inducible NTCP expression

**Figure 1:**
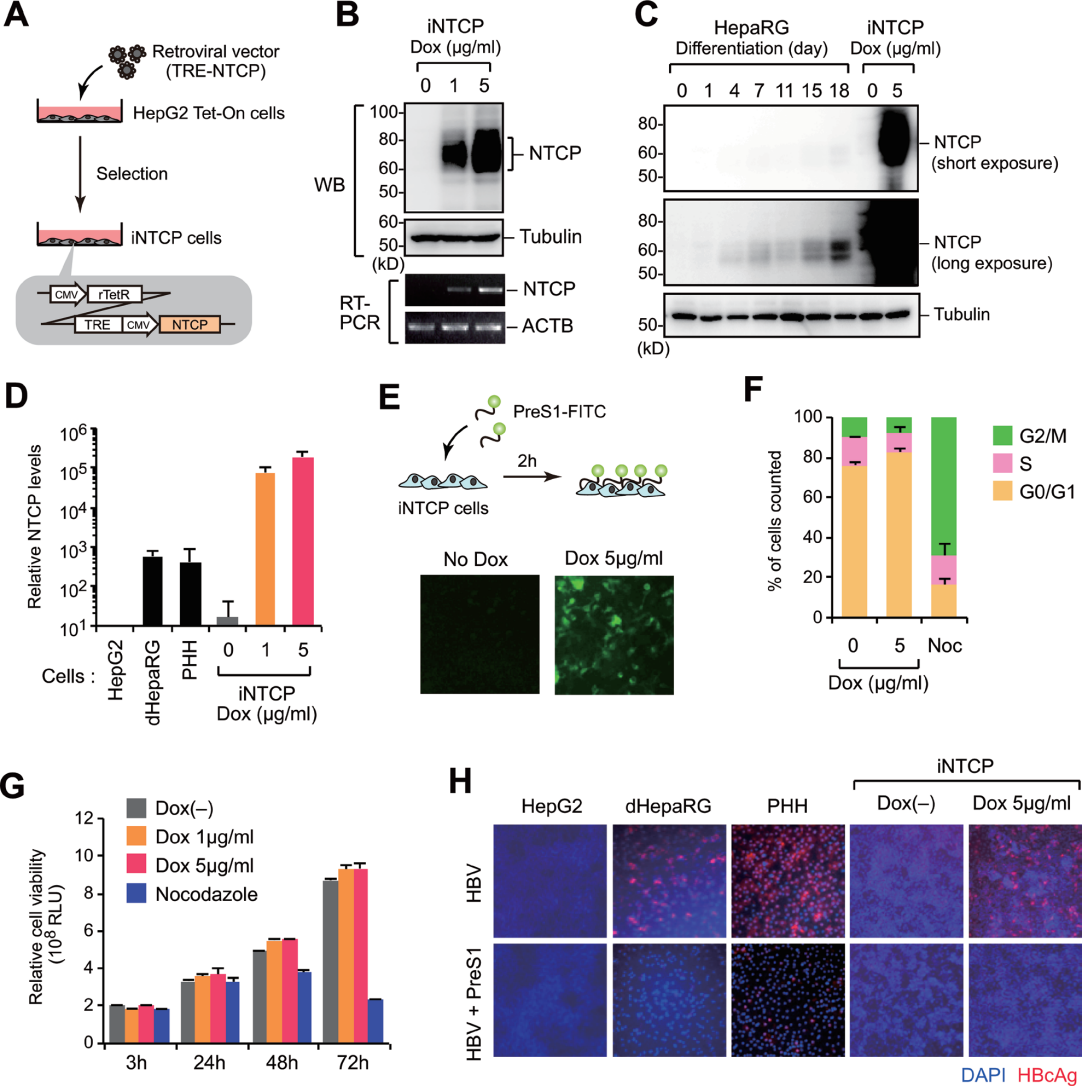
Preparation of HBV-permissive HepG2 cells with inducible NTCP expression (**A**) Generation of iNTCP cells. A HepG2 Tet-On parental cell line was transduced with a retroviral vector encoding the NTCP gene fused to a tetracycline-responsive element (TRE) and then selected with puromycin. (**B**) iNTCP cells were treated with doxycycline (Dox) at the indicated concentrations for 24 hours. NTCP expression was then verified by western blotting (upper panels) or RT-PCR (lower panels). (**C, D**) NTCP expressions in indicated cells were determined by western blot (C) and qPCR (D). (**E**) PreS1-binding assay. iNTCP cells pretreated with 5 μg/ml of Dox for 24 hours were incubated with 400 nM FITC-conjugated PreS1 peptide for two hours before fixation and microscopy. (**F**, **G**) Induced NTCP does not affect cell proliferation. Cell cycle and cell proliferation assays of iNTCP cells. Cells were treated with Dox for 72 hours before cell cycle analysis. Nocodazole (Noc; 100 nM) was used as a control for inducing cell cycle arrest. (**H**) High susceptibility to HBV infection of iNTCP cells. Indicated cells were infected with HBV for 16 hours in the presence or absence of PreS1 peptide, cultured for six days, then stained with anti-HBcAg antibody (red) and DAPI (blue). iNTCP cells were treated with Dox for 24 hours before infection as well as during infection.

Endogenous NTCP expression is rarely detectable in hepatoma cell lines such as HepG2 and Huh7, and these cells are not susceptible to HBV infection. Therefore, we generated the iNTCP cell line, a HepG2-based cell line harboring a tetracycline-inducible human *NTCP* gene (Figure 1A). Treatment of the iNTCP cell line with the tetracycline analogue doxycycline (Dox) caused expression of NTCP in a Dox-dependent manner, and NTCP expression was 20- to 100-fold higher than endogenous expression in differentiated HepaRG cells and primary hepatocytes, as revealed by western blot and quantitative reverse transcription-PCR (Figure 1B–1D). To determine whether Dox-induced NTCP protein localized to the plasma membrane, we performed a PreS1 peptide binding assay. We found that the PreS1 peptide detected Dox-treated iNTCP cells but not untreated cells, indicating the presence of NTCP on the cell surface (Figure 1E). Consistent with a previous report [6], a western blot of NTCP showed major bands of 60–80 kD that were shifted to a single band of 30–40 kD after treatment with peptide N-glycosidase (PNGase), implying that NTCP was modified by N-glycosylation (Supplementary Figure 1A). Although NTCP expression has been reported to affect cell proliferation [17], iNTCP cells showed no differences in cell cycle progression or cell expansion with or without Dox treatment (Figure 1F, 1G). We subsequently examined the susceptibility of iNTCP cells to HBV infection. At an MOI of 6000 GEq/cell, iNTCP cells showed high susceptibility to HBV infection (~80% infected) without DMSO treatment (Figure 1H, Supplementary Figure 1B). This infection was significantly inhibited by PreS1 peptide treatment (Figure 1H), which indicates that HBV infection was NTCP-mediated. Taken together, these results show that Dox-induced NTCP proteins are exposed on the cell surface and functionally interact with PreS1.

### Development of monoclonal antibody specifically targeting cell-surface NTCP

**Figure 2:**
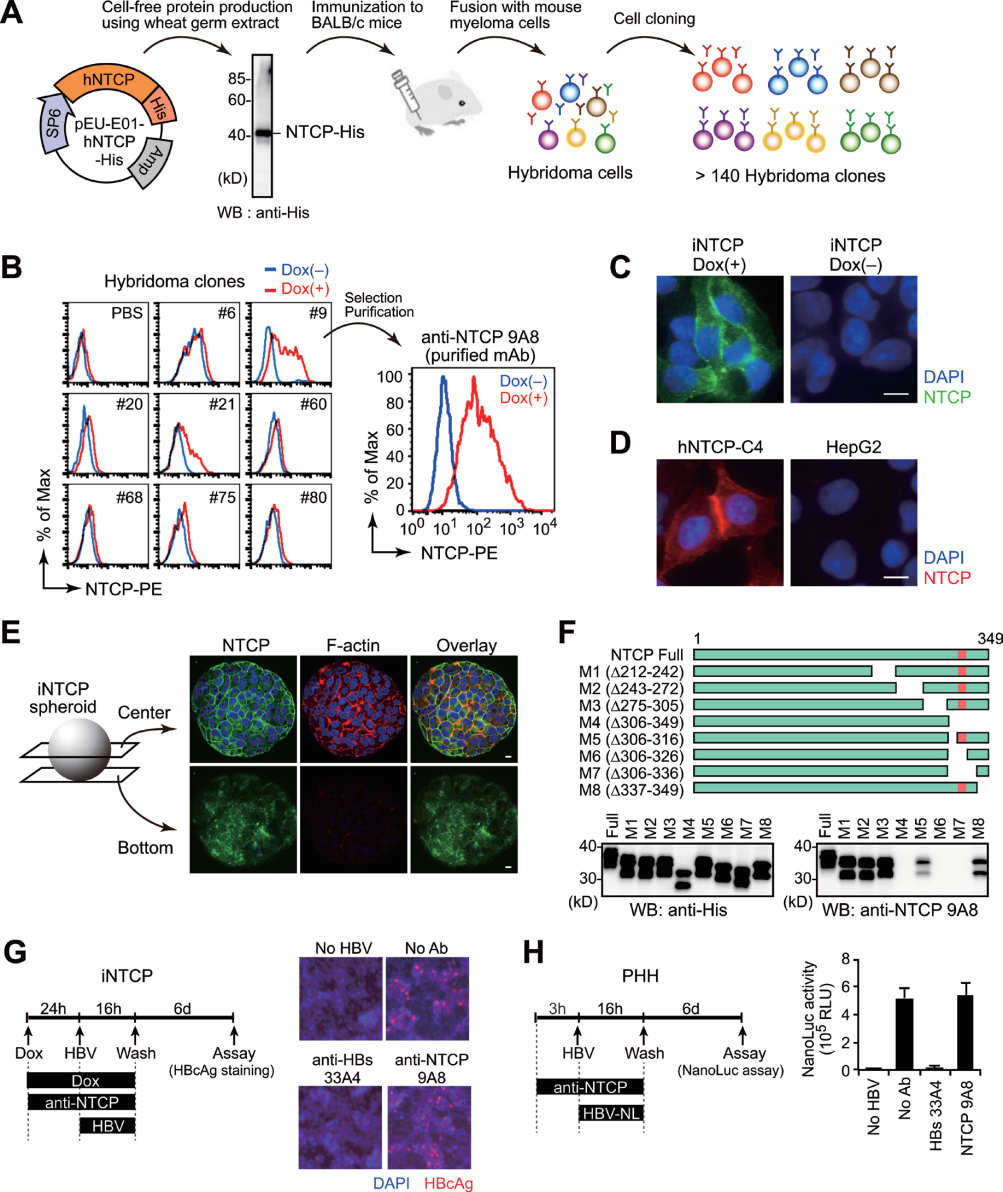
Development of a monoclonal antibody specifically targeting cell-surface NTCP (**A**) Schematic illustration of anti-NTCP mAb generation. Using a wheat germ cell-free protein production system, large amounts of NTCP were synthesized with high solubility. Following mouse immunization, we established over 140 hybridoma clones. (**B**) Selection of 9A8 clones producing anti-NTCP mAb. Culture supernatants of hybridoma clones were used as primary antibodies for flow cytometry analysis of Dox-treated (red line) or -untreated iNTCP cells (blue line). (**C–E**) Immunofluorescence staining of cell-surface NTCP by 9A8 mAb on iNTCP cells (C), HepG2-hNTCP-C4 cells (D), and iNTCP-derived spheroid (E). Scale bars: 10 μm. (**F**) Epitope mapping of 9A8 mAb. Recombinant wild-type or partially truncated NTCP proteins tagged with His were generated using wheat germ extracts and subjected to western blotting using anti-His or 9A8 antibodies. The predicted epitope of 9A8 mAb is shown in pink. (**G**, **H**) 9A8 mAb fails to inhibit HBV infection. iNTCP cells (G) and primary human hepatocytes (H) were infected with HBV or its reporter virus (HBV-NL) respectively, in the presence of 9A8 mAb. Anti-HBs mAb (clone 33A4, which recognizes the PreS1 domain) was used as a control. Viral infectivity was determined by intracellular HBcAg staining (G) or NanoLuc activity (H) of infected cells.

Although the above results suggest that NTCP proteins localize on the cell surface, this could not be directly demonstrated due to the lack of suitable antibodies for flow cytometry or immunofluorescence microscopy analysis. Therefore, we generated a monoclonal antibody (mAb) for this purpose. Because recombinant NTCP protein tends to form insoluble aggregates, we utilized the wheat germ cell-free system, which has been shown to have advantages for the production of membrane proteins [18, 19]. The synthesized NTCP proteins were purified and used to immunize mice, and more than 140 hybridoma clones were established (Figure 2A). Using a flow cytometer-based screening assay with Dox-treated and untreated iNTCP cells, we identified a hybridoma clone producing anti-NTCP mAb, clone 9A8 (Figure 2B). The 9A8 mAb could recognize endogenous NTCP in differentiated HepaRG cells (Supplementary Figure 2). We performed immunofluorescence microscopy using the 9A8 mAb in various cell lines and cell-surface NTCP was clearly observed (Figure 2C, 2D). Because the three-dimensional organoid culture recapitulates cell-cell interactions and recent studies have indicated some advantages of hepatoma organoids in hepatitis virus infection [20], we investigated the localization of NTCP in hepatoma organoids. We embedded iNTCP cells in hydrogel before performing immunofluorescence microscopy with the 9A8 mAb to visualize NTCP. NTCP localization was widespread in the membrane of internal cells as well as on the surface of the organoid (Figure 2E). Epitope mapping using recombinant NTCP mutants revealed that the 9A8 mAb recognizes amino acids 317–326 of NTCP (Figure 2F). To test whether the 9A8 antibody can inhibit HBV infection, we pretreated iNTCP cells and primary human hepatocytes with 9A8 mAb and subsequently infected cells with wild type HBV and HBV encoding a luciferase reporter gene (HBV-NL) [21]. The 9A8 mAb failed to inhibit HBV infection (Figure 2G, 2H), suggesting that the interaction between 9A8 mAb and NTCP neither blocks HBV-host cell interaction nor causes downregulation of NTCP from the cell surface.

### Identification of compounds that downregulate cell-surface NTCP

**Figure 3:**
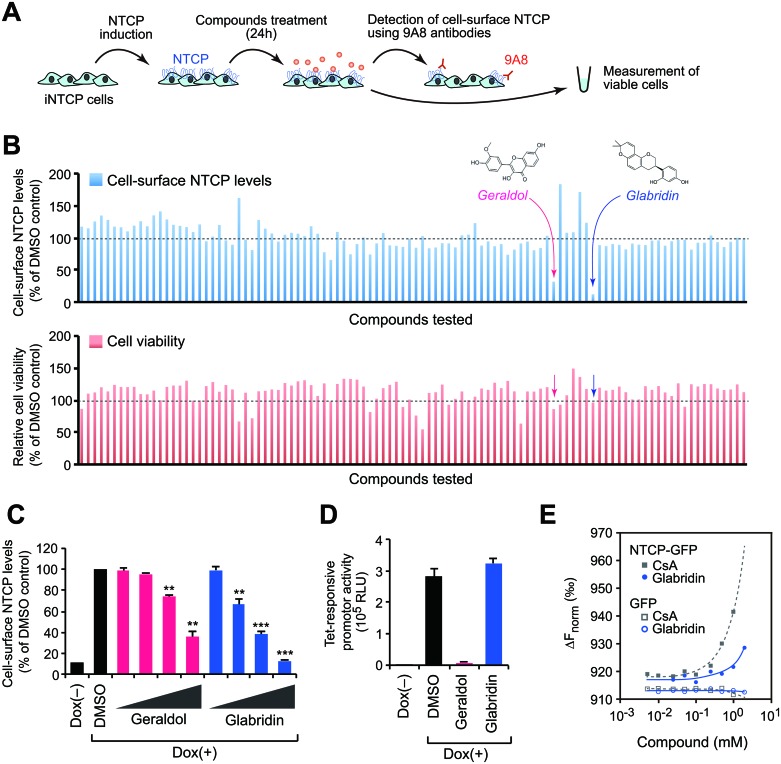
Identification of compounds that downregulate cell-surface NTCP (**A**) Schematic representation of the screening procedure. (**B**) Cell-surface NTCP expression (upper) and cell viability (lower) of cells treated with candidate compounds. (**C**) Flow cytometry analysis. Cell-surface NTCP expression of iNTCP cells treated with indicated compounds (5, 10, 25, and 50 μM) for 24 hours. ^**^P < 0.01, ^***^P < 0.001, two-tailed unpaired *t*-test. (**D**) Glabridin does not affect the tetracycline-responsive promoter activity. U2OS cells stably expressing a Dox-inducible luciferase gene were treated with indicated compounds (50 μM) for 24 hours, then subjected to a luciferase assay. (**E**) Biomolecular binding of compounds to NTCP. Microscale thermophoresis analysis was performed to determine the interaction between glabridin and recombinant NTCP-GFP or GFP. A known NTCP-binding compound, cyclosporin A (CsA), was used as a positive control. The y-axis represents normalized fluorescence intensity.

Because the 9A8 mAb binds NTCP but does not interfere with its localization and function, it can be used to screen for antiviral compounds that modulate the level of cell-surface NTCP. Briefly, iNTCP cells were treated with 102 different low-molecular-weight chemical compounds from a library derived from natural plant extracts for 24 hours and we subsequently quantified cell viability and the amount of cell-surface NTCP using the 9A8 mAb (Figure 3A). We identified two compounds, geraldol and glabridin, that could decrease the amount of cell-surface NTCP without observable cytotoxicity (Figure 3B). Flow cytometry analysis using the 9A8 mAb demonstrated that both geraldol and glabridin decrease the levels of cell-surface NTCP in a dose-dependent fashion (Figure 3C), but further analysis revealed that geraldol negatively regulates the tetracycline-responsive promoter activity (Figure 3D). Microscale thermophoresis analysis of the biomolecular interaction [22] revealed that glabridin could weakly but directly interact with NTCP (Figure 3E). Therefore, we focused on glabridin for further functional analyses.

### Glabridin induces caveolar endocytosis of NTCP

**Figure 4:**
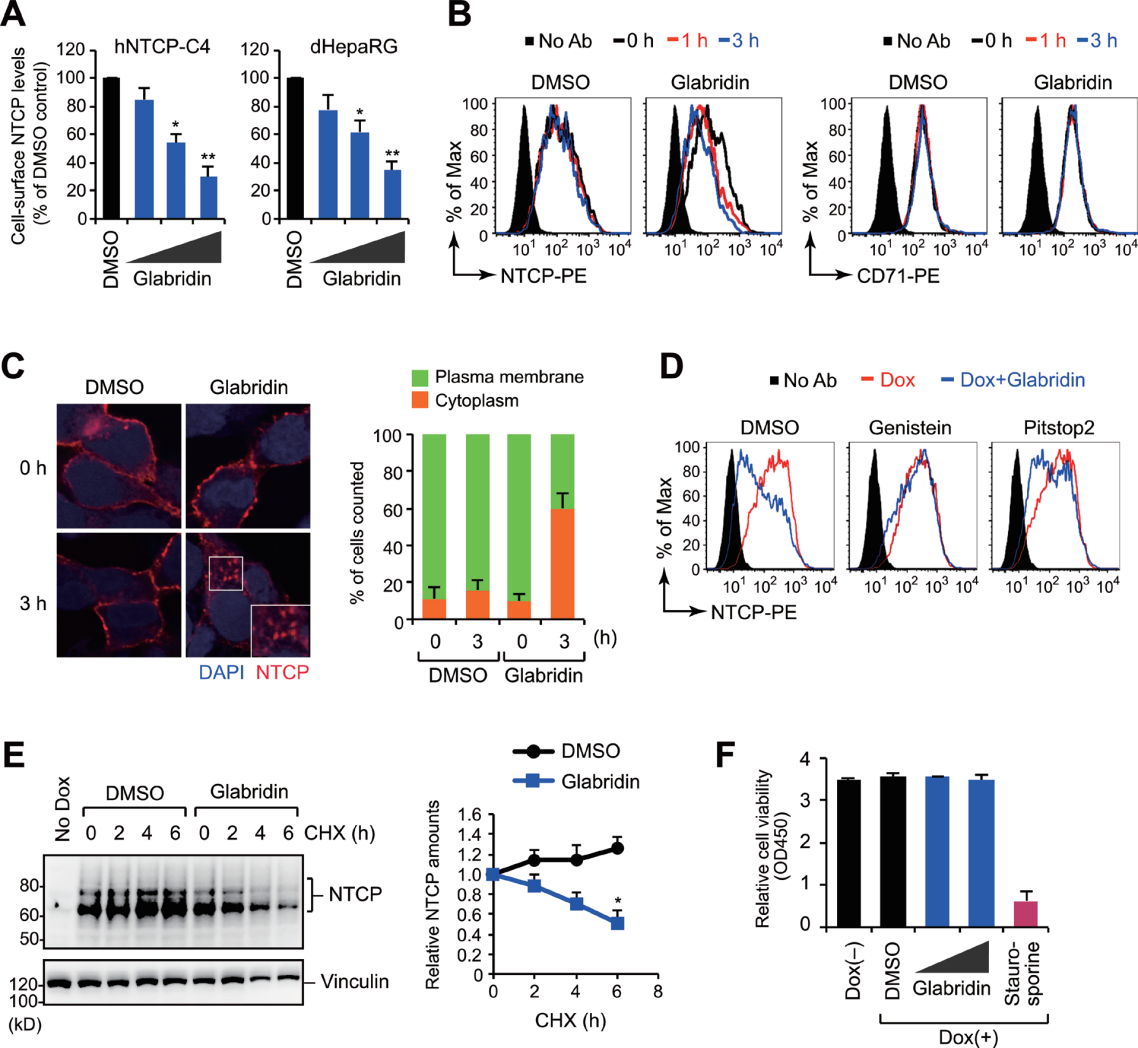
Glabridin induces caveolar endocytosis of NTCP (**A**) Flow cytometry analysis. Cell-surface NTCP expression of cells treated with glabridin (10, 25, and 50 μM) for 24 hours. ^*^P < 0.05, ^**^P < 0.01, two-tailed unpaired *t*-test. (**B**) Rapid down-regulation of cell-surface NTCP by glabridin. iNTCP cells were treated with glabridin (50 μM) for designated intervals and were analyzed by flow cytometer after staining with 9A8 mAb (left) or anti-transferrin receptor antibody (right). (**C**) Internalization of NTCP by glabridin. Subcellular localization of NTCP was determined by immunofluorescence microscopy using the 9A8 mAb. (**D**) Flow cytometry analysis of iNTCP cells treated concurrently with glabridin (50 μM) and genistein (a caveolar endocytosis inhibitor, 50 ng/ml) or Pitstop2 (a clathrin-mediated endocytosis inhibitor, 30 μM). (**E**) Glabridin leads to NTCP degradation. iNTCP cells were treated with cycloheximide (CHX) in the presence or absence of glabridin (50 μM). ^*^P < 0.05, two-tailed unpaired *t*-test. (**F**) Glabridin does not exhibit cytotoxicity in effective concentrations. Cell viability assay of iNTCP cells treated with glabridin (25 and 50 μM) for 48 hours. Staurosporine (1 μM) was used as a control for inducing cell death.

Flow cytometry analysis showed that glabridin downregulated the amount of cell-surface NTCP in HepG2-hNTCP-C4 [23] and HepaRG cells with IC_50_ values of 28 μM and 34 μM, respectively (Figure 4A). Notably, treatment with 50 μM glabridin had no effect on the level of NTCP mRNA (Supplementary Figure 3A), suggesting that glabridin affects NTCP protein rather than NTCP gene expression. Consistently, treatment with glabridin rapidly (within three hours) modulated the membrane localization of NTCP, but not that of the transferrin receptor (Figure 4B). Similar results were obtained with cell-surface biotinylation analysis in which only cell-surface proteins were purified and detected by western blotting (Supplementary Figure 3B). Immunofluorescence microscopy using the 9A8 mAb showed that in glabridin-treated cells, NTCP mainly accumulated in the cytoplasm (Figure 4C). To test the role of endocytosis in NTCP localization, we treated cells concurrently with glabridin and inhibitors of clathrin-mediated or caveolar endocytosis. We found that genistein, a specific inhibitor of caveolar endocytosis, completely disrupted NTCP internalization (Figure 4D), suggesting that glabridin causes the internalization of NTCP through caveolar endocytosis.

Because internalized membrane proteins are typically trafficked to degradation or recycling pathways, we next performed a cycloheximide chase assay. Interestingly, NTCP protein levels were relatively stable in control cells, but the half-life of NTCP was significantly shorter in glabridin-treated cells (Figure 4E). This suggests that glabridin reduces the amount of NTCP on the cell surface by promoting its endocytosis and subsequent intracellular degradation. Previous reports have indicated that glabridin induces apoptosis in certain cancer cells [24], but we observed no negative effect on cell viability under our experimental conditions (Figure 4F).

### Glabridin inhibits HBV infection in primary human hepatocytes

**Figure 5:**
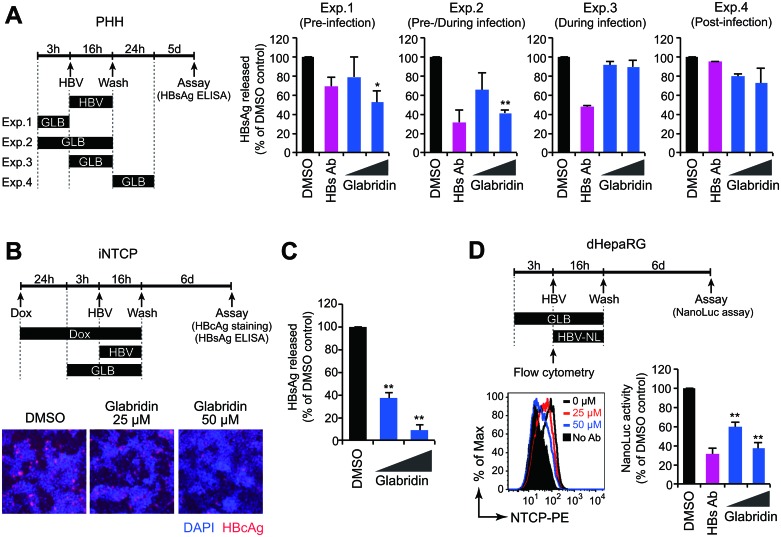
Glabridin inhibits HBV infection in primary human hepatocytes (**A**) Time-of-drug-addition experiments. Primary human hepatocytes were infected with HBV in the presence of glabridin for 3–24 hours according to the procedure schematized at the left. Culture supernatants were subjected to the HBsAg ELISA six days after infection. ^*^P < 0.05, ^**^P < 0.01, two-tailed unpaired *t*-test. (**B**, **C**) Glabridin suppresses HBV infection in iNTCP cells. iNTCP cells were infected with HBV in the presence of glabridin (25 and 50 μM). Cells were pretreated with Dox (5 μg/ml) one day before infection, and with glabridin three hours prior to infection. Cells and culture supernatants were subjected to intracellular HBcAg staining (B) and quantification of HBsAg released (C). ^**^P < 0.01, two-tailed unpaired *t*-test. (**D**) Glabridin suppresses HBV infection in HepaRG cells. Differentiated HepaRG cells were infected with HBV-NL in the presence of glabridin (25 and 50 μM). Infectivity was determined by assaying luciferase activity six days after infection. Cell-surface NTCP expression of HepaRG cells treated with glabridin for three hours was measured by flow cytometry. ^**^P < 0.01, two-tailed unpaired *t*-test.

We next assessed the impact of glabridin on HBV infection in hepatocytes. Time-of-addition experiments demonstrated that 50 μM glabridin inhibited HBV infection when added early in infection but became less effective when added later (Figure 5A), suggesting that it acts at an early stage of the viral life cycle. Indeed, when glabridin was added to HBV-producing cells, it did not affect the amounts of core-associated viral DNA and/or RNA (Supplementary Figure 4). Consistent with these observations, iNTCP cells treated with glabridin three hours prior to infection showed a significant reduction in the percentage of infected cells and the amount of HBsAg secretion (Figure 5B, 5C). We performed a parallel analysis with differentiated HepaRG cells and confirmed that glabridin blocked HBV infection and downregulated endogenous NTCP with an IC_50_ value of 33 μM (Figure 5D). These results suggest that glabridin inhibits HBV infection by removing NTCP from the cell surface.

### Glabridin suppresses bile acid uptake by depleting cell-surface NTCP

**Figure 6:**
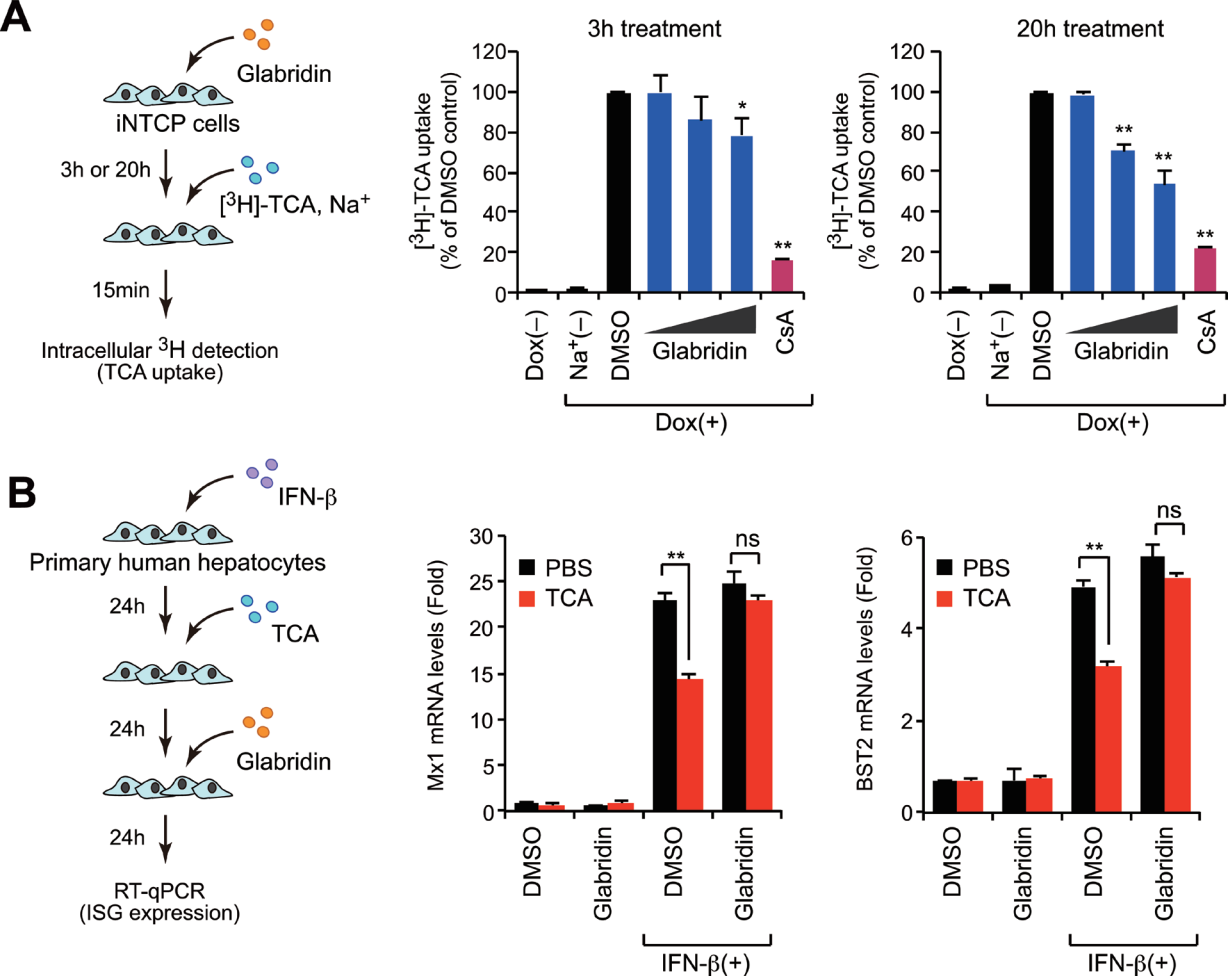
Glabridin suppresses bile acid uptake by depleting cell-surface NTCP (**A**) Glabridin inhibits taurocholate uptake. iNTCP cells were treated with glabridin (10, 25, and 50 μM) for three or 20 hours. After incubation with [^3^H]-taurocholate (TCA) for 15 minutes, cells were washed and intracellular radioactivity was quantified. Cyclosporin A (CsA, 10 μM) was used as a positive control in this assay. ^*^P < 0.05, ^**^P < 0.01, two-tailed unpaired *t*-test. (**B**) Glabridin counteracts bile acids to promote innate immune signaling. Primary human hepatocytes were sequentially treated with IFN-β (100 U/ml), TCA, and glabridin (50 μM), as shown in left panel. The expression of representative ISGs, including Mx1 and BST2, was quantified using qPCR. ns, not significant; ^**^P < 0.01, two-tailed unpaired *t*-test.

NTCP is a transporter for bile acid uptake. We investigated the effect of glabridin on NTCP-mediated uptake of taurocholate (TCA) in a sodium-containing condition and found that glabridin reduced TCA uptake in a dose- and time-dependent manner (Figure 6A). A previous study has shown that NTCP-mediated bile acid transport affects the expression of IFN stimulatory genes (ISGs) in primary human hepatocytes [15], so we investigated whether glabridin counteracts this function of bile acid. Primary human hepatocytes were treated with type I IFN and TCA in the presence or absence of glabridin. Treatment with IFN upregulated various ISG proteins including Mx1 and BST2, which are known to inhibit HBV replication [25–28], while concurrent treatment with TCA reduced this effect (Figure 6B), in accordance with previous observations [15]. Interestingly, the addition of 50 μM glabridin partially cancelled the effect of TCA in ISG expression (Figure 6B). These findings suggest that glabridin enhances the innate immune response by suppressing bile acid uptake in hepatocytes through the downregulation of cell-surface NTCP.

## DISCUSSION

In this study, we generated iNTCP cells, which have high NTCP expression and high susceptibility to HBV infection, and also developed a monoclonal antibody (mAb) that recognizes cell-surface NTCP. Using these tools, we identified glabridin as a compound that inhibits HBV infection by downregulating levels of its entry receptor, NTCP. Although primary hepatocytes express NTCP at low levels for the uptake of bile acids, endogenous NTCP in hepatocellular carcinoma cell lines is not sufficient to achieve successful infection with HBV *in vitro*. Hepatocellular carcinoma cell lines stably expressing NTCP have been created, and exhibited increased susceptibility to HBV infection [29], but it has been shown that continuous NTCP expression can induce cell cycle arrest at the G0/G1 phase [17], indicating that ectopic NTCP expression may be unfavorable for the long-term culture. To overcome these issues, we created the iNTCP cell line, featuring inducible NTCP expression, using the third-generation tetracycline-inducible gene expression system in HepG2 cells. Upon transient treatment with tetracycline derivatives, this cell line exhibits high expression of cell-surface NTCP and becomes highly susceptible to HBV infection without any observable cell cycle arrest. This unique feature of our newly developed cell line makes it a useful tool for further biological analysis of NTCP in hepatic cells.

We created a new mAb recognizing cell-surface NTCP by using a wheat germ cell-free protein production system to synthesize full-length recombinant NTCP protein, which was then used to immunize mice. Generally, the quality of an antibody is determined largely by the antigen used for immunization. Preparation of immunogens derived from highly insoluble membrane proteins is challenging, and NTCP tends to be insoluble in conventional cell expression systems due to its multiple transmembrane domains. There are several methods for preparing antigens derived from insoluble proteins, including use of synthetic peptides containing the predicted immunogenic epitope of extracellular domains [30], but synthetic peptides are structurally linear and do not recapitulate the native structural features of membrane proteins. By contrast, the wheat germ cell-free protein production system utilizes a eukaryotic translation system to synthesize structurally intact and biologically active proteins similar to those expressed in mammalian cells [31, 32]. This approach enabled us to create a mAb capable of detecting a spatial antigen within the region of NTCP exposed on the cell surface. Our work demonstrates the advantage of the cell-free system in the production of proteins with multiple transmembrane domains [18, 19].

The three-dimensional structure of NTCP protein has not been well characterized and the number of transmembrane domains is controversial at present. Several groups have predicted that NTCP has 7–9 transmembrane domains and that the C-terminus of NTCP is located in the cytoplasm [33, 34]. We found that our 9A8 mAb recognizes amino acids 317–326 of NTCP on intact cells without membrane permeabilization, implying that this epitope is possibly exposed to the extracellular space. More precise structural biological studies should be carried out to elucidate the topology of the NTCP protein.

Immunofluorescence microscopy experiments using our 9A8 antibody revealed that NTCP localized on the plasma membrane, frequently at cell-cell contact sites. This localization was more obvious in a hepatocyte organoid culture system, suggesting that the localization of NTCP may depend on cell polarity. Because the three-dimensional culture system recapitulates cell polarity, it is useful for analyzing the function of NTCP in sodium taurocholate transport as well as HBV infection. Recent studies demonstrated that the three-dimensional culture system is suitable for analyzing polarized HBV transmission [35, 36]. It is not well established whether NTCP plays a role in viral transmission in polarized cells, and our newly developed 9A8 mAb may be useful for pursuing this intriguing question.

Several compounds have been shown to target NTCP. Recent studies demonstrated that antagonists of retinoic acid receptor Ro41-5253 [37] or the cytokine interleukin-6 [38] could reduce NTCP expression. A previous report also indicated that the green tea extract epigallocatechin-3-gallate (EGCG) also inhibited HBV entry into primary human hepatocytes [39]. Interestingly, EGCG induced endocytosis of NTCP from the plasma membrane followed by protein degradation. In the current study, we found that glabridin, a compound from licorice extract, also causes NTCP receptor downregulation. Although the precise mechanisms are unclear, our findings suggest that glabridin directly binds NTCP and causes its internalization by caveolar endocytosis. By estimating protein turnover using a cycloheximide chase assay, we were able to infer that glabridin promotes the intracellular degradation of NTCP. Interestingly, glabridin is orally bioavailable and is known to rapidly accumulate in the liver [40–42]. Since IC_50_ value of glabridin for inhibiting of HBV entry is relatively high (~40 μM), this compound might have little translational benefit in HBV research. However, the data obtained from our study demonstrates the potential scope of our current methodology in drug discovery for HBV therapeutics.

It is possible that the internalization of NTCP could inhibit its transporting activity thereby causing unfavorable effects on hepatocytes. However, people with I223T or S267F mutations in the NTCP gene show decreased surface expression and transporting activity of NTCP, but there have been no reports of serious diseases resulting from these mutations to date [43, 44]. The frequent occurrence of physiological downregulation of NTCP is also notable. This downregulation is typically controlled by transcription factors, such as HNF4α, under cholestasis conditions [45]. Furthermore, recent papers have shown that NTCP is the main transporter for conjugated bile acids into the liver, but other auxiliary transporters, such as OATP, may compensate when NTCP is absent [46, 47]. Although these reports hint that transient downregulation of NTCP might not induce immediate adverse effects on the liver, the effect should be further investigated in *in vivo* models to determine whether NTCP inhibition is a reasonable anti-HBV drug target.

In conclusion, we identified as glabridin as a natural compound capable of inhibiting HBV infection by impairing viral entry into host cells, with an additional effect of enhancing the antiviral immune response. Furthermore, our data demonstrate that our newly developed cell line and antibody will serve as powerful tools for drug discovery targeting HBV entry and for exploring molecular mechanisms underlying HBV spread.

## MATERIALS AND METHODS

### Cells and compounds

To generate iNTCP cells, a HepG2 Tet-On Advanced Cell (Clontech) parental cell line was transduced with a retroviral vector encoding the NTCP gene fused to a tetracycline-responsive element, then was selected with puromycin (1 μg/ml), and cultured with DMEM (Wako) supplemented with 10% FBS. Unless otherwise indicated, iNTCP cells were treated with doxycycline (Sigma-Aldrich) for 24 hours before experiments. The iNTCP spheroid was made using the 3-D Life Dextran-CD Hydrogel SG kit (Cellendes) and cultured for seven days on a chamber slide (Thermo Fisher Scientific). Primary human hepatocytes (PXB-cells) were purchased from PhoenixBio. HepaRG cells were purchased from Biopredic International and differentiated according to the manufacturer's instructions. The chemical compound library from natural plant extracts was obtained from Tokiwa Phytochemical. Nocodazole, cyclosporin A, genistein, and Pitstop2 were purchased from Sigma-Aldrich. Glabridin, staurosporine, and IFN-β were obtained from Wako.

### Generation of anti-NTCP antibody

A Protemist XE robotic protein synthesizer (CellFree Sciences) was used for the generation of full length NTCP and its truncated derivatives as previously described [48, 49]. Immunization of BALB/c mice with recombinant NTCP and generation of hybridoma cells producing anti-NTCP antibody were performed as previously described [48]. Purification of antibodies in the culture supernatant of the hybridoma clones was performed by centrifugation at 8,000 rpm for 15 minutes and elution with AcroSep Hyper DF columns (Pall). Samples were then concentrated using Amicon Ultra filters (Merck Millipore). Immunoglobulin characterization was carried out using the IsoStrip mouse monoclonal antibody isotyping kit (Roche).

### Microscopic analysis

Cells were fixed with 4% paraformaldehyde, blocked with 10% normal goat serum (Thermo Fisher Scientific), and stained with either anti-NTCP mAb (clone 9A8) or anti-HBcAg polyclonal antibody (Dako) and Alexa Fluor-conjugated secondary antibodies (Thermo Fisher Scientific). Alexa Fluor 594-conjugated phalloidin (Thermo Fisher Scientific) was used for F-actin staining. For intracellular staining, cells were permeabilized with 0.5% Triton X-100 before blocking. For the PreS1-peptide binding assay, iNTCP cells pretreated with 5 μg/ml Dox for 24 hours were incubated with 400 nM FITC-conjugated PreS1 peptide (the first 59 amino acid residues of small HBs domain fused with N-terminal myristoyl group and C-terminal FITC) at 37° C for two hours. Cells were then washed with PBS and fixed with 4% paraformaldehyde. Microscopic imaging was performed with an FV1000-D confocal laser scanning microscope (Olympus) or BZ-9000 fluorescence microscope (Keyence).

### Flow cytometry

Cells were detached with 5 mM EDTA in PBS and fixed with 4% formaldehyde before incubation with either anti-NTCP (9A8) or anti-transferrin receptor (Genetex) antibodies at 4° C. Cells were then stained with PE-conjugated secondary antibody and analyzed using a FACSCanto II instrument (BD Biosciences). For the drug screen, cells were treated with candidate compounds (50 μM) 24 hours before analysis. Data were analyzed with FlowJo software (TreeStar).

### HBV preparation and infection

Wild-type HBV was derived from the supernatants of HepG2.2.15 cells, which were stably transfected with a complete HBV genome. HBV reporter viruses (HBV-NL) were produced by transient transfection of HepG2 cells with pUC1.2-HBV/NL and pUC-HBV-D, as previously described [21]. The collected supernatants were filtered through a 0.45-μm filter (Merck Millipore), and concentrated approximately 100 times using a PEG Virus Precipitation kit (BioVision). Cells were infected with wild-type HBV at a concentration of 5,000 genome equivalents per cell in the presence of 4% PEG8000 for 16 hours. Alternatively, cells in a 96-well plate were inoculated with 5 μl of HBV-NL in the presence of 4% PEG8000 for 16 hours. HBV-infected cells were cultured in fresh medium for an additional 5–6 days and their infectivity was determined by intracellular HBcAg staining or extracellular HBsAg quantification, as previously described [28]. The infectivity of HBV-NL was quantified using the Nano-Glo Luciferase System (Promega), according to the manufacturer's instructions.

### Western blotting

Samples in SDS loading buffer were loaded onto 10% polyacrylamide gels, electrophoresed, and blotted onto PVDF membranes (Merck Millipore), as previously described [28]. Membranes were probed with primary antibodies and horseradish peroxidase-conjugated secondary antibodies (GE Healthcare). For protein degradation analysis, cycloheximide (100 μg/ml) was added 2–6 hours before harvesting cells. The primary antibodies used were as follows: anti-NTCP, anti-vinculin, anti-α-tubulin (Sigma-Aldrich) and anti-His (Genetex). Detected proteins were visualized using a FluorChem digital imaging system (Alpha Innotech). Band analysis was performed with ImageJ software (National Institutes of Health).

### Gene expression analysis

Messenger RNA extraction and subsequent cDNA synthesis was performed using Trizol reagent (Thermo Fisher Scientific) and ReverTra Ace (Toyobo), respectively, according to each manufacturer's instructions. Gene expression was then analyzed by qPCR using SYBR Premix Ex Taq II (Takara) and a CFX96 Real-Time PCR Detection System (Bio-Rad). The primer pairs used were 5′-ggacttcgagcaagagatgg-3′ and 5′-agcactgtgttggcgtacag-3′ for ACTB; 5′-atggaggcccacaacg cgtctgccc-3′ and 5′-cagaaggtggagcaggtggtcatcac-3′ for NTCP; 5′-ggctgtttaccagactccgaca-3′ and 5′-cacaaa gcctggcagctctcta-3′ for Mx1; and 5′-tctcctgcaaca agagctgacc-3′ and 5’-tctctgcatccagggaagccat-3’ for BST2.

### Microscale thermophoresis analysis

Recombinant NTCP-GFP and GFP proteins were incubated with different concentrations of compounds in 50 mM potassium phosphate buffer (pH 7.0) containing 100 mM NaCl, 0.2% BSA, and 0.005% DDM (n-Dodecyl-β-D-maltopyranoside) for one hour at room temperature. Samples were loaded on Monolith NT.115 Standard Treated Capillaries (Nano Temper Technologies) and analyzed with a Monolith NT.115 Blue Red microscale thermophoresis instrument.

### Cell cycle and cell viability assays

Cell cycle analysis was performed with a Tali image-based cytometer (Thermo Fisher Scientific). Cell viability was measured with Cell Counting Kit-8 (Dojindo) or CellTiter-Glo assay (Promega) according to the manufacturer's instructions.

### NTCP transport assay

Cells were treated with [^3^H]-taurocholic acid (TCA) in a sodium-free or sodium-containing buffer at 37°C for 15 minutes to allow TCA uptake into cells. Cells were washed and intracellular radioactivity was measured using a liquid scintillation counter.

### Statistical analysis

All graphs represent means and standard deviations. The statistical significance of differences between two groups was tested using a two-tailed unpaired *t* test with Prism 6 software (GraphPad).

## SUPPLEMENTARY MATERIALS FIGURES



## Abbreviations

NTCP: sodium taurocholate cotransporting polypeptide
HBV: hepatitis B virus
CHB: chronic hepatitis B
IFN: interferon
HBeAg: hepatitis B e antigen
HBsAg: hepatitis B surface antigen
mAb: monoclonal antibody
Dox: doxycycline
PNGase: peptide N-glycosidase
TCA: taurocholate
ISG: interferon stimulatory gene
