# Supplementary material for: Development of a cell-based assay to identify hepatitis B virus entry inhibitors targeting the sodium taurocholate cotransporting polypeptide
Source: Oncotarget. 2018 May 4;9(34):23681–94. doi: 10.18632/oncotarget.25348 (PMC5955094; doi:10.18632/oncotarget.25348)
Supplement: Supplementary file 1 [file oncotarget-09-23681-s001.pdf]

Development of a cell-based assay to identify hepatitis B virus entry inhibitors targeting the sodium taurocholate cotransporting polypeptide

SUPPLEMENTARY MATERIALS

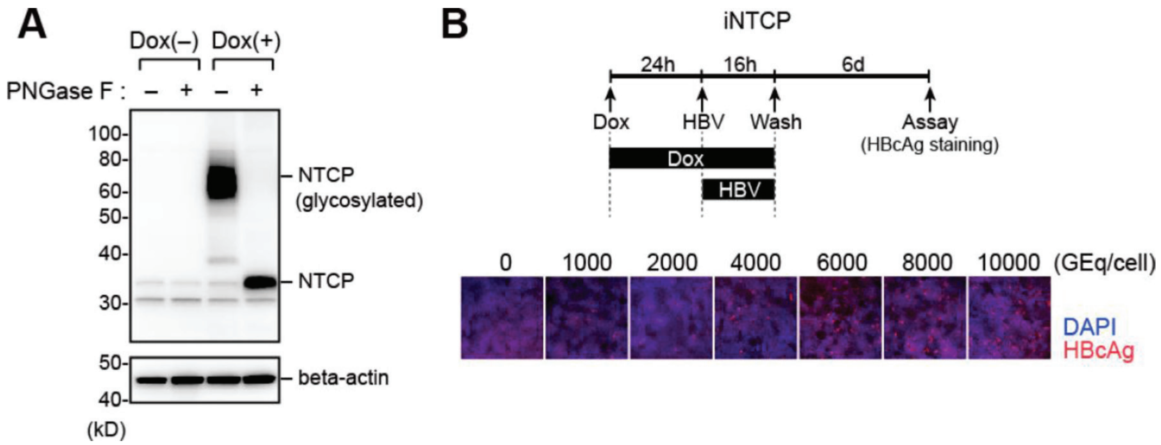

Supplementary Figure 1: Characterization of iNTCP cells. (**A**) Induced NTCP proteins were highly glycosylated. iNTCP cells were treated with doxycycline (Dox) for 24 hours. Cell lysates were incubated with or without PNGase F (5 units/μl) at 37° C for one hour before western blotting. (**B**) Optimization of HBV inoculum dose in Dox-treated iNTCP cells. Cells were infected with HBV at the indicated MOI for 16 hours, cultured for six days, then stained with anti-HBcAg antibody (red) and DAPI (nuclei, blue).

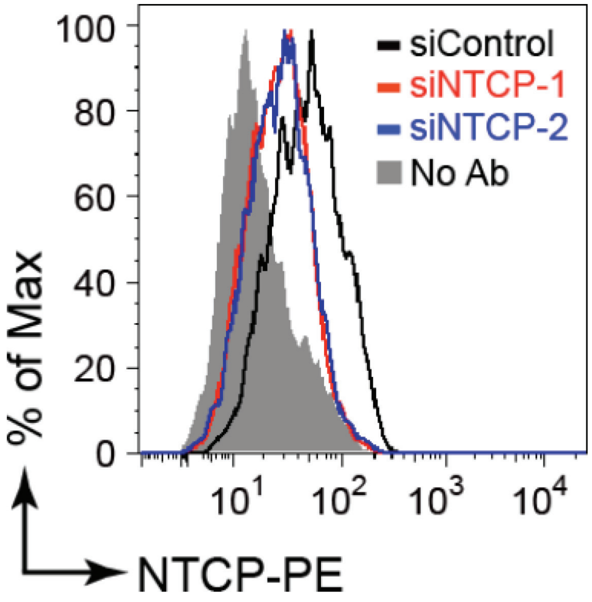

Supplementary Figure 2: The 9A8 mAb detects endogenous NTCP in HepaRG cells. Differentiated HepaRG cells were transfected with control siRNA or NTCP-targeted siRNAs (QIAGEN). Cells were stained using 9A8 mAb and subjected to flow cytometry 24 hours after transfection.

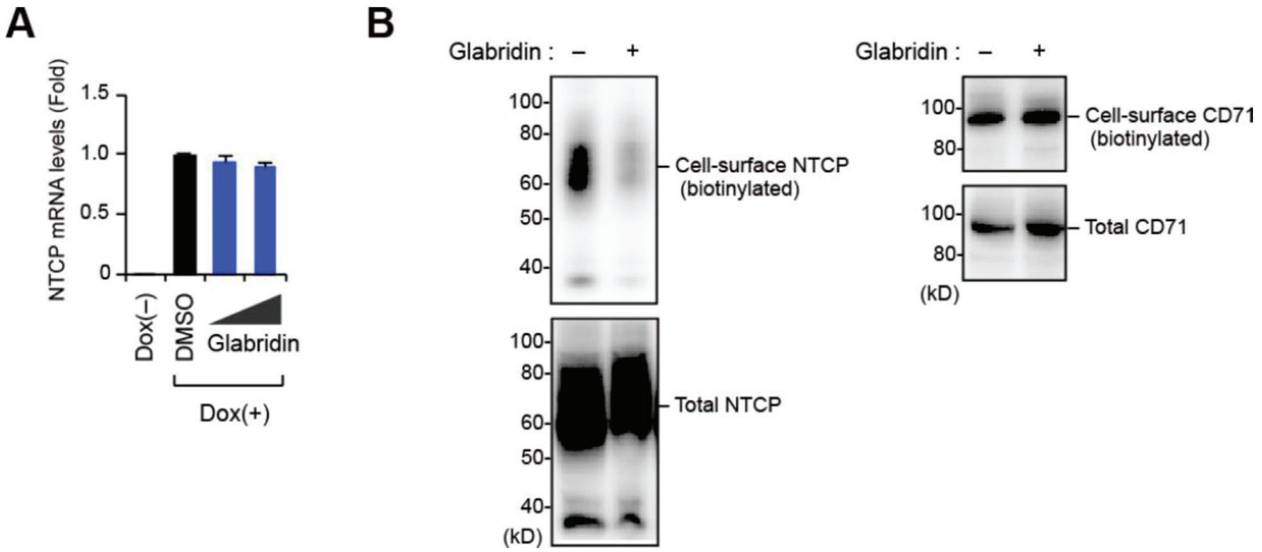

Supplementary Figure 3: Glabridin acts posttranslationally to reduce cell-surface NTCP. (**A**) Glabridin does not affect the level of NTCP mRNA. iNTCP cells were treated with glabridin (25 and 50 μM) for 24 hours. Expression of NTCP mRNA was quantified using qPCR. (**B**) Surface biotinylation analysis of iNTCP cells treated with glabridin (50 μM) for three hours. Cell surface proteins were biotinylated, precipitated, and eluted using a Cell Surface Protein Isolation Kit (Thermo Fisher Scientific). Cell lysates (total proteins) and eluates (biotinylated cell-surface proteins) were then subjected to western blotting.

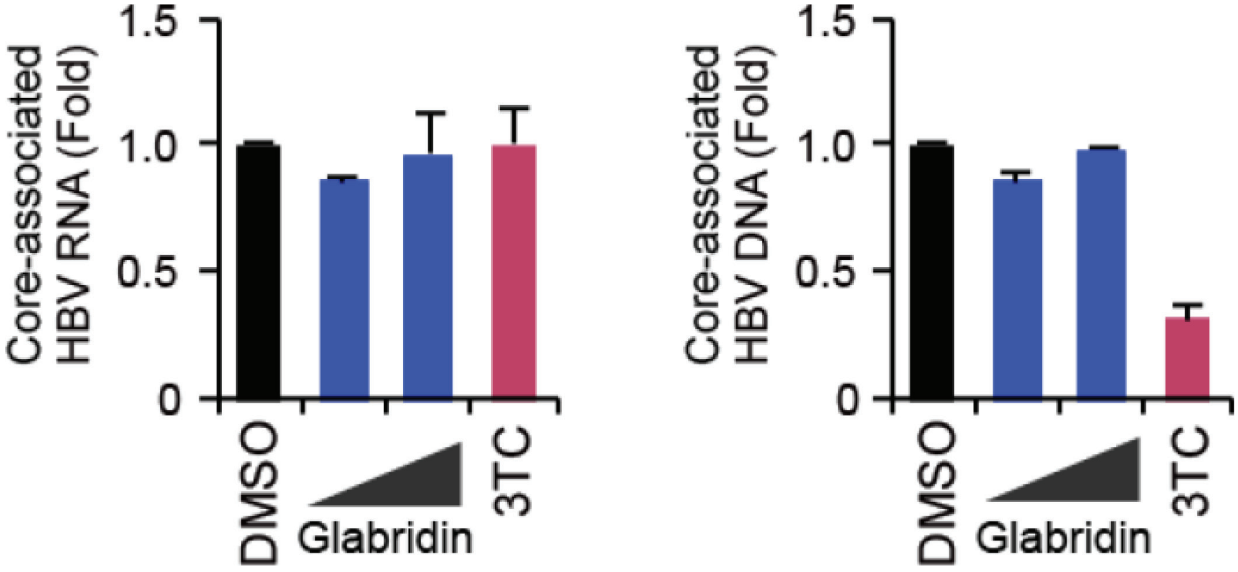

Supplementary Figure 4: Glabridin has no observable effects on the amounts of capsid-associated viral genome. HepG2.2.15.7 cells stably producing HBV were treated with glabridin (25 and 50 μM) for 24 hours and the capsid-related viral genome was subsequently quantified. Lamivudine (3TC, 1 μM) is a reverse transcriptase inhibitor and used as a control.
